# Population Structure of Atlantic Mackerel (*Scomber scombrus*)

**DOI:** 10.1371/journal.pone.0064744

**Published:** 2013-05-31

**Authors:** Teunis Jansen, Henrik Gislason

**Affiliations:** DTU AQUA *-* National Institute of Aquatic Resources, Technical University of Denmark, Charlottenlund Castle, Charlottenlund, Denmark; University of Canterbury, New Zealand

## Abstract

Atlantic mackerel (*Scomber scombrus*) occurs on both sides of the north Atlantic and has traditionally been grouped into 5 spawning components, some of which were thought to be isolated natal homing stocks. Previous studies have provided no evidence for cross Atlantic migration and no or weak support for isolated spawning components within either side of the North Atlantic. We question the de-facto accepted hypothesis of isolation between spawning components on the basis of spawning and age distribution data. The spawning intensities, proxied by larval abundances, are negatively correlated between the North Sea and Celtic Sea, which indicates that the two spawning components may be connected by straying individuals. This finding is based on unique larvae samples collected before the collapse of North Sea component, thus showing that the exchange is not a recent phenomenon due to the collapse. The analyses of old as well as more recent age distributions show that strong year classes spread into other areas where they spawn as adults (“twinning”). Our findings are in accordance with the lack of solid evidence for stock separation from previous analyses of tagging data, genetics, ectoparasite infections, otolith shapes, and blood phenotypes. Because no method has been able to identify the origin of spawning mackerel unequivocally from any of the traditional spawning components, and in the light of our results, we conclude that straying outweighs spatial segregation. We propose a new model where the population structure of mackerel is described as a dynamic cline, rather than as connected contingents. Temporal changes in hydrography and mackerel behavior may affect the steepness of the cline at various locations. The new interpretation of the population structure of Atlantic mackerel has important implications for research, assessment and management.

## Introduction

Mackerel (*Scomber scombrus*) is one of the most abundant and widely distributed migratory fish species in the North Atlantic [1]. Mackerel live their entire life in the pelagic environment. Early life stages (eggs and young larvae) drift passively with the currents until they start undertaking vertical migrations. Young juveniles begin to migrate horizontally, and mature adult individuals perform extensive horizontal migrations between overwintering, spawning and feeding areas [2].

### Traditional Spawning Components

**Figure 1:**
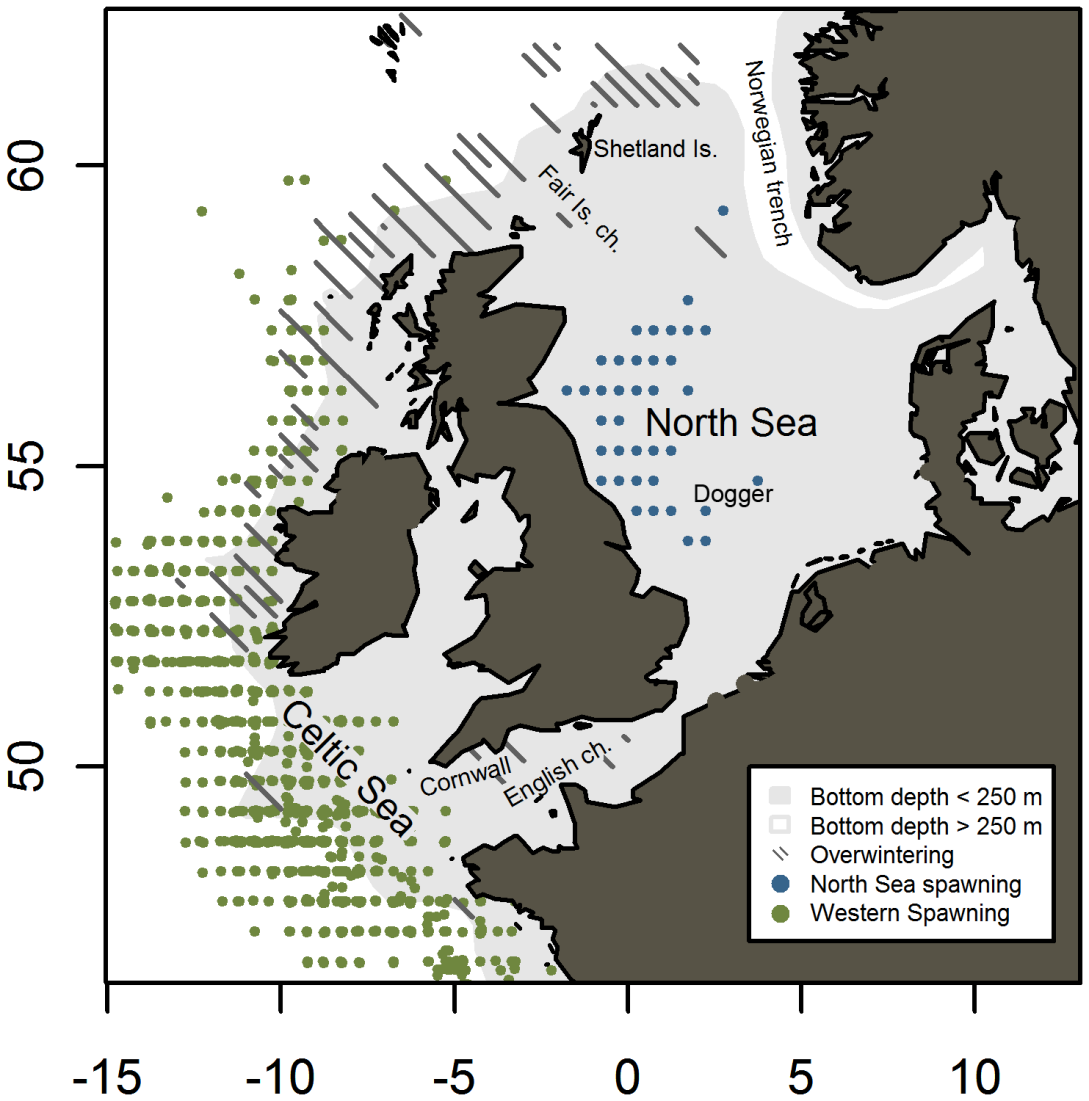
Mackerel populations and distribution around the North-west European shelf. Continental shelf marked in grey (bottom depth <250 m). Spawning areas indicated by dots. Each dot marks an observation of 50+ eggs m^−2^ day^−1^ (data from international mackerel egg surveys in the North Sea 2002–2011 [73]–[75] and western areas 1977–2007 (ICES WGMEGS)). Stripes mark the distribution of mackerel before spawning (based on commercial catch data in January-March 1985–2010) [52].

In the North East Atlantic (NEA) mackerel spawn from the Mediterranean Sea in the south to the Faroe Islands in the North and from Hatton Bank in the West to Kattegat in the East (Figure 1). Spawning starts in January in the Mediterranean Sea, February off the Portuguese coasts and ends in July north of Scotland and in the North Sea [3]. While spawning varies locally from day to day [4], [5], it seems to form one large spatiotemporal continuum on the larger scale. However, relatively low levels of spawning in the English and Fair Isle channels separates the main spawning areas in the North Sea from the western areas along the continental shelf edge [6]. Despite the lack of complete spatial or temporal separation, NEA mackerel have traditionally been divided into three distinct entities, namely the Southern, Western and North Sea spawning components [1], [7]. This excludes the less well known Mediterranean spawners.

In the North West Atlantic (NWA) mackerel spawning has not been mapped by synoptic surveys. The mackerel are fished from North Carolina in the South to Newfoundland and Labrador in the North [8]. The presence of a southern and a northern spawning contingent off the US and Canadian coasts has been suggested [8], [9].

Despite numerous studies, there is very limited knowledge about the isolation and mixing between these 5 entities (herein called spawning components) and they remain weakly defined. No previous study has discussed the application of the metapopulation concept in relation to Atlantic mackerel.

### Contingents and Metapopulations

Populations where life-cycle patterns can be categorized into multiple components or contingents are often referred to as a *metapopulations* [10]. A life-cycle pattern can be defined by recurring and persistent migration and dispersion processes that link the sequential habitats used by the different life stages [11]. A population can thus be characterized by a single pattern i.e. panmixia, or by a diversity of patterns i.e. as a metapopulation consisting of multiple contingents. While panmixia is simple to define as “random mating within the population” (www.Wikipedia.org), the metapopulation is more challenging to define. Levins first defined the metapopulation as: *a population of local populations which were established by colonists, survive for a while, send out migrants, and eventually disappear. The persistence of a species in a region depends on the rate of colonization successfully balancing the local extinction rate* [12]. Hanski & Simberlo later relaxed the definition to its most simple form: *presence of discrete local breeding populations connected in exchange of individuals* [13]. Smedbol et al. revised the numerous definitions and usages and found that the concept was being increasingly used, but also misused [10]. They underlined the importance of contingents having nontrivial probabilities of experiencing extinction during the lifespan of the metapopulation.

Large marine fish stocks, like cod and herring, consist of a diversity of life-cycle patterns. They are thus often referred to as metapopulations consisting of contingents. The contingents are usually contained within persistent oceanographic structures that ensure larval retention and/or control migration of adults [14]. However, the contingents can be more or less connected by straying of individuals through dispersal in the larval, juvenile, and/or adult phase [14], [15]. Life-cycle patterns are not necessarily genetically inherited and their persistence could be explained by phenotypic plasticity and social behavior [11], [16]. The differences in life-cycle patterns are often reflected in phenotypic characters [14] due to the different environmental conditions that each contingent experience. In some cases, low contingent connectivity on an evolutionary time scale, have allowed for minor genetic differentiation [17], [18]. Some authors have argued that species like cod, does not form true metapopulations because the extinction-criteria are not likely to be fulfilled for these species due to the high mixing (straying) rates [10]. However, for all species that are found on both sides of the Northern Atlantic, and where fish from each side do not occasionally interbreed, contingents could become extinct on either side of the Atlantic. Subsequent recolonization could then take place in warmer times, where the two sides are not isolated by cold water masses.

The key questions regarding metapopulations and contingent connectivity are: Are there more than one closed life-cycle pattern, i.e. contingents? Is there a possibility of extinction of at least one contingent? Are there strays that switch from one life cycle to another? Does the breeding success of stray mackerel counter the isolating effect of natal homing, leading to a prevention of genetic differentiation?

### Phenotypic Plasticity, Homing and Genetic Diversity

Tagging data and genetic analysis strongly supports a separation of mackerel on the eastern and western side of the Atlantic. Out of the approximately 1 million mackerel tagged in the East Atlantic, none have been reported as recaptured in the western Atlantic [19]–[21], and differences in mitochondrial DNA in mackerel from the two sides have been identified [22].

However, within both the eastern and western Atlantic distribution areas most studies have failed to find significant signs of stock separation in phenotypic, genotypic or life-cycle patterns. Studies in the NEA which have tried to identify mackerel spawning components from phenotypic characters, such as juvenile growth patterns in otoliths [23], [24], age compositions [25], length at age [25], protein polymorphism [26], [27], nematode (*Anisakis simplex*) [25] and tapeworm (*Grillotia smarisgora*) infection rates [28], have been unable to demonstrate significant differences. Unfortunately, these studies were based on individuals from the respective spawning areas that were *not* all in the process of spawning (*i.e.* ripe/running). These studies may thus have included mackerel from several discrete components, due to the swimming capabilities of mackerel. One fish was marked in the channel off the South-East coast of England and recaptured 1200 nm away off Shetlands after just 13 days [29]. As mackerel swim continuously day and night [30], this corresponds to approximately 4 knots, which is well below the maximum swimming speed measured in-situ on schools [31]. After spawning, some mackerel from the south-western areas of the NEA migrate into the North Sea before spawning in the North Sea has ceased [20]. Consequently, conclusions on natal homing and the existence of multiple components cannot be drawn from these studies.

The studies in the NEA that were based on spawning individuals, also found no difference when examining ectoparasite infections [32] and blood phenotypes [33]. Otolith shapes differ across the Atlantic and can to some extent be used to identify the origin of mackerel (60–87% successfully identified) [34], however no difference was found between the North Sea and the western components of the NEA (Jansen unpubl. analysis of 652 mackerel otoliths). Although statistically significant differences were found within the NWA, the distributions of shape parameters were not sufficiently discrete to allow for actual identification purposes [34]. Most recently, significant differences in juvenile growth patterns have been detected within the western component in the East Atlantic [35]. The latter study compared growth data (fish length) with latitude and found that southern juvenile mackerel attained a greater length than those from further north. Examination of juvenile otolith rings on adult spawning mackerel revealed a similar significant relationship between growth and latitude for adult mackerel spawning between latitudes 44°N (Bay of Biscay) and 54°N (west of Ireland). This means that a significant proportion of a given year class returned to spawn at higher latitudes, than other individuals from the same year class that were hatched at lower latitudes. These findings thus rejected panmixia by indicating spatially segregated life cycles among North East Atlantic mackerel [35].

Tagging experiments have unfortunately not been designed to follow the homing and mixing of the three different putative components as the maturity stage has not been recorded during tagging and recapture. However, incomplete mixing between mackerel tagged in the Celtic Sea during spawning time and mackerel tagged in the North Sea in August after the spawning season (i.e. a mixture of migrants) [36], supports rather than rejects some sort of separation.

Genetic studies on the eastern side of the Atlantic have so far been inconclusive, and whether the balance between spatial segregation and mixing has allowed for genetic differentiation within the populations on each side of the Atlantic remains to be thoroughly examined. Three studies of gene variants did *not* find that the samples from the NEA grouped into the expected clades (spawning components) [22], [26] and (Pers.Comm. Frode Lingaas, 21 Sept. 2011). However, a different statistical analysis of the mitochondrial DNA allele frequencies from one of the studies separated the 3 samples from the western area from the rest in the NEA [22]. However, this analysis was based on relatively few samples (3+3+4) with few individuals (22+17+16) and it did not account for differences between year classes. The weak support for genetic differentiation in this study may therefore have been generated on an ecological time scale rather than on an evolutionary time scale. Genetic studies on the mackerel in the NWA are similarly inconclusive [8]; while genetic differences have been suggested by studies on the polymorphism of some proteins [37]–[39], more recent phylogenetic and molecular variance analysis did not reveal genetic differences between the northern and the southern component [40].

Atlantic mackerel thus display isolated and different life-cycle patterns across the Atlantic Ocean. On each side, there seem to be a diversity of spatiotemporal life cycle patterns, but no method has successfully been able to unequivocally identify the origin of spawning mackerel from any of the traditional spawning components. The tendency for spatial segregation within one component, does on the other hand demonstrate that mackerel exhibit the necessary behavioral element that act towards closure of spatiotemporal life cycle patterns on a more localized scale. Assuming that the tendency for spatial segregation within the spawning migration is a global mackerel phenomenon, we can direct our focus on mixing processes that counter the differentiating effect of spatial segregation.

### Straying between Spawning Components

Mixing between components can be caused by passive drift or active migration in any life stage. In this study, we focus on adult strays, i.e. mackerel that originate from one area but spawn in another.

A new index of North Sea mackerel spawning stock size have recently been published based on a unique historic material of mackerel larvae catches from the Continuous Plankton Recorder (CPR) survey and a new approach to CPR data modelling [41], [42]. The new index showed substantial interannual variability in the period of high abundance from the early 1950s to late 1960s [41]. The interannual variation clearly exceeds the potential effects of recruitment and mortality, because mackerel does not mature until 2–3 years of age [1], and can live for over 20 years [29]. Other migratory pelagic species, such as herring, are also structured into natal homing spawning stocks. These stocks are not isolated as straying between the stocks has been documented [11], [43]. Similar connectivity, between the mackerel stocks in the North Sea and the western areas, is a potential explanation for the observed interannual variation of North Sea mackerel.

To test the hypothesis of mixing between the North Sea and western spawning component, we compared spawning stock sizes (proxied by densities of early larvae) in the North Sea and the Celtic sea. If the two stocks are indeed separate, then the historical development of stock sizes should most likely differ, but more important: The interannual variability should *not* be negatively correlated as this would indicate that mackerel can switch spawning area preference from year to year. We furthermore investigated potential environmental influences on the spawning migration (switching of areas) in this period.

Straying in the adult phase can also be traced using natural tags, such as strong and contrasting year classes. The demography of NEA mackerel seems to be dominated by strong year classes in some periods such as the 1950s–1960s in the North Sea [44], [45], early 1980s and in the latter decade [1]. From mid 1980s to 2000, recruitment was relatively constant [1]. Age distributions may therefore serve as natural tracers of mackerel in some periods. In this study I focus on two strong year classes: The 1969 year class from before the collapse in the North Sea and the 2005 year class that mainly recruited from the area west of Scotland.

## Materials and Methods

### Mackerel Spawning Stock Size Index

**Figure 2:**
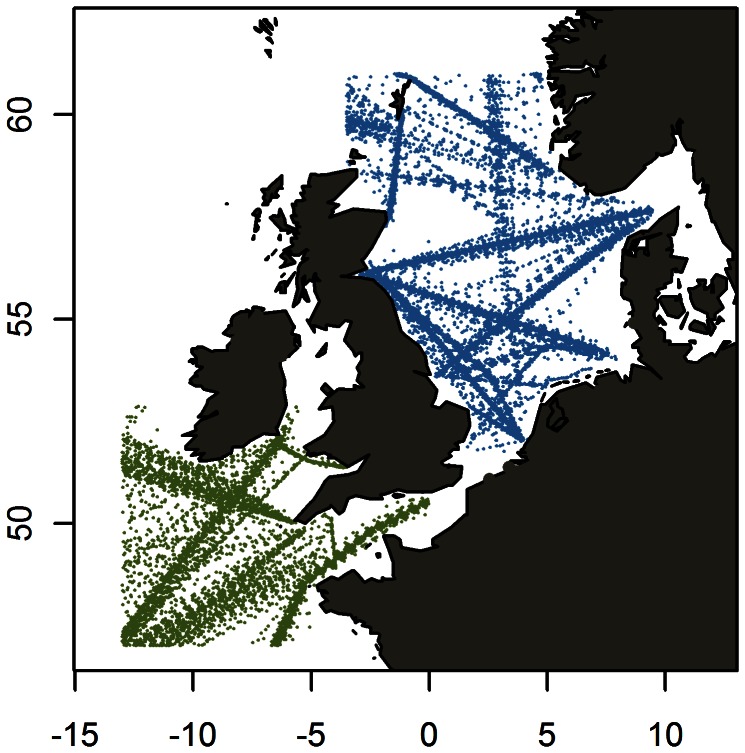
Continous plankton recorder samples from 1955–1974 in the studied areas.

Mackerel larvae from Continuous Plankton Recorder (CPR) surveys from 1951 to 1974 covering the central spawning areas in the North and Celtic Seas (51–61°N, 3.5°W-9.5°E and 47–53°N, 13°W-0°E) were kindly provided by SAHFOS [46]. The CPR were towed by ships of opportunity at speeds in the range 15–20 knots and at an approximate depth of 10 m. Water entered the recorder through an aperture of 1.62 cm^2^, and was filtered through a continuously moving band of silk with an average mesh size of 270 µm. The plankton was fixed in formalin. The silk band was divided into samples representing 10 miles of tow, equivalent to approximately 3 m^3^ of filtered seawater. Methods of counting and data processing are described by [47], [48]. The dataset consisted of 2,870 larvae observations in 21,906 samples, widely spread through the spawning season in the central spawning areas of the North and Celtic Seas (Figure 2 and [46]).

A log-gaussian cox process model [46] was fitted to the larvae observations in each of the two spawning areas and used to generate time series of annual larval indices. Unlike the deterministic raising algorithms often applied to CPR data, this state-of-the-art statistical technique accounts for both catchability as well as spatial and temporal autocorrelation. Model documentation is published in [41], [49] and followed in this study, except that thermocline depth was not applied as a fixed effect as no data were available for the Celtic Sea. To test the effect of this alteration on the time series of annual values, we compared the North Sea index modelled with and without thermocline depth.

The comparison of larval indices in the two areas was restricted to 1955–1974, because the Celtic Sea area was inadequately surveyed before 1955 and spawning in the North Sea decreased dramatically during the 1970s. To test the first null-hypothesis of *uncorrelated stock size trends*, we used running means as proxies for stock size trends. The more years spanned by the running mean, the less data points. To ascertain that the test result would be robust to the chosen number of spanned years, we tested with 3 and 5 year intervals. To test the second null-hypothesis of *uncorrelated interannual variability*, we compared the indices from the North and Celtic Seas for the period 1958–1966. No distinct trends were apparent in this period and the spawning in the North Sea was at a high level, making it more likely that fluctuations could be detected in the western area if mackerel switched between spawning areas from year to year. This makes the period from the change in CPR methodology (1958) to the initiation of the Norwegian purse seine fishery (∼1966) ideal for testing the second and most important null-hypothesis. However, due to the low number of observations in this period (9 years), the second null-hypothesis was also tested by correlating detrended annual index values, calculated as 3-year running means minus the annual value. This was done for the whole period 1955–1974.

We furthermore tested if environmental parameters important for adults, eggs and larvae could have driven the migration in the period where spawning and the signal-to-noise ratio in the larvae index were at their highest. A multivariate linear model was used to explore potential causes for the long term temporal variability in spawning in the North Sea. The North Sea larvae index as provided by [41] was again used as a proxy for annual spawning intensity. The study period was expanded to 1951–1966, which corresponds to the period where commercial fisheries found plenty of mackerel in the North Sea. The period begins in 1951 with the beginning of the good years for the Dutch fishery (>10 kt/year) and ends in 1966 with the large Norwegian fishery (>500 kt/year). Unlike the previous analysis, we included data from both before and after the change in CPR methodology in 1958, because the North Sea index did not show a related and distinct drop in index values as was seen in the Celtic Sea index. However, this was only possible for the model runs without the Zooplankton parameter, since the zooplankton timeseries starts in 1958.

The initial model included the following candidate predictors:

- Sea surface temperature (Spring_SST) in the spawning area and early spawning season. Spring_SST was included because it has been shown to be correlated with spawning distribution in the western areas [50] and timing of spawning in the North Sea [51].
- Winter temperature (Winter_T) in the shelf edge current where mackerel overwinters. Winter_T was included because it affects the distribution prior to the spawning migration with a possible knock-on effect into the spawning season [52].
- Sea surface salinity (Spring_SSS) in the spawning area and early spawning season. Spring_SSS was included because salinity has been shown to be related to spawning in a more coarse long term analysis of larvae abundance. This relation may not be directly causal, but salinity could indicate certain water masses that are preferred by mackerel [42].
- Zooplankton concentration (Zoo) in the whole North Sea, as this is important food for larvae, juveniles and adults [2].

Interactions were omitted due to the limited number of data points. For the same reason, we also tested for correlation between the response variable and each single predictor variable separately.

Predictor variables were Z-transformed. The *corvif* function of the AED R-package was used to calculate Variance Inflation Factors (VIF). VIFs are indicators of collinearity. The predictor variables were sufficiently independent to be used in the same model fit, if the VIFs were >3 [53]. Inspection of Auto Correlation Function (ACF) plots (not shown) revealed no temporal autocorrelation in the response variable. Model-building was done “backwards” by sequentially removing insignificant (p>0.05) terms.

The analysis of potential environmental effects was further expanded by mapping the correlation between the larval index and the parameter that was found to be significant in the first model. Multiple time series of the parameter, one for each area (2°×4° rectangles), was calculated. A map was then produced to visualize the strength of the correlation between each of these time series and the larval index.

### Temperature, Salinity and Zooplankton

Time series of annual Spring_SST and Spring_SSS values were estimated as the average of monthly temperature and salinity in April-June in the area 56–62°N, 0–4°W. The 5937 observations were obtained from ICES hydrographic database [54] and originated from samples taken by CTD/bottles/underway/pump/moorings at less than 10 m of depth. A modelled time series of Winter_T was obtained from [52].

Zooplankton data from CPR surveys from 1958 to 1974 were obtained from the SAHFOS database as abundance by species by sample. Biomass by sample was calculated using the mean dry weight by species from [55]. Mean zooplankton concentration (g dry weight/m^3^) by year were calculated as a simple average of all samples in the North Sea (50–60°N 4°W-8°E) in the peak spawning season (June). Biomass was used instead of abundance because mackerel in all life stages are size selective feeders and prefer larger calanoid copepods over smaller cyclopoid copepods [56]–[60]. The CPR is known to under-sample in some situations [55]. We did not correct for under-sampling because it mostly affects smaller species [55].

Modelling and correlation tests were performed in R version 2.12.1 with the “stats”, “nlme”, and “sp” packages [61].

### Recruitment

Four time series of recruitment in the North Sea were used to identify strong year classes. i) Catch rates of 4 year old mackerel in the 1955–1961 year classes in the Dutch trawl fishery assuming these were fully recruited to the fishery [44]. ii) Number of recruits of the year classes 1962–1970 from a landings and tagging based assessment [45]. iii) Catch rates of first winter juveniles from the international bottom trawl survey (IBTS) in the first quarter of 1968 - 1979 from [62]. iv) Catch rates of first winter juveniles in the first quarter of 1973 - 2010 from the ICES DATRAS database (http://datras.ices.dk). The four recruitment indices were thus not on the same absolute scales. In order to visualize the strong year classes within the same plot, we standardized the values in each data set to the mean of each time series. The last time series of catch rates were further downscaled by an arbitrary factor of 0.2.

Catch Per Unit Effort (CPUE, numbers hour^−1^) of juvenile mackerel at the age of zero was obtained from international bottom trawl surveys in October-December. CPUE was used as reported to and compiled by ICES WGWIDE.

### Age Distributions

The fraction of the commercial catch consisting of the 1969 year class of mackerel was obtained from [63] for the area between the Outer Hebrides and Cape Butt by month from 1974–1979.

Age distributions in commercial catches in Jan-Mar and Jul-Sep 2010 by ICES division were obtained from [1]. Divisions with insignificant fisheries (<500,000 mackerel) in Jan-Mar were excluded.

## Results

### Spawning

Removal of thermocline depth from the original larvae model [41] did not affect the *temporal* aspect of the larval model as we found the North Sea indices modelled with and without thermocline depth to be highly positively correlated (p<0.001, R^2^ = 0.996).

**Figure 3:**
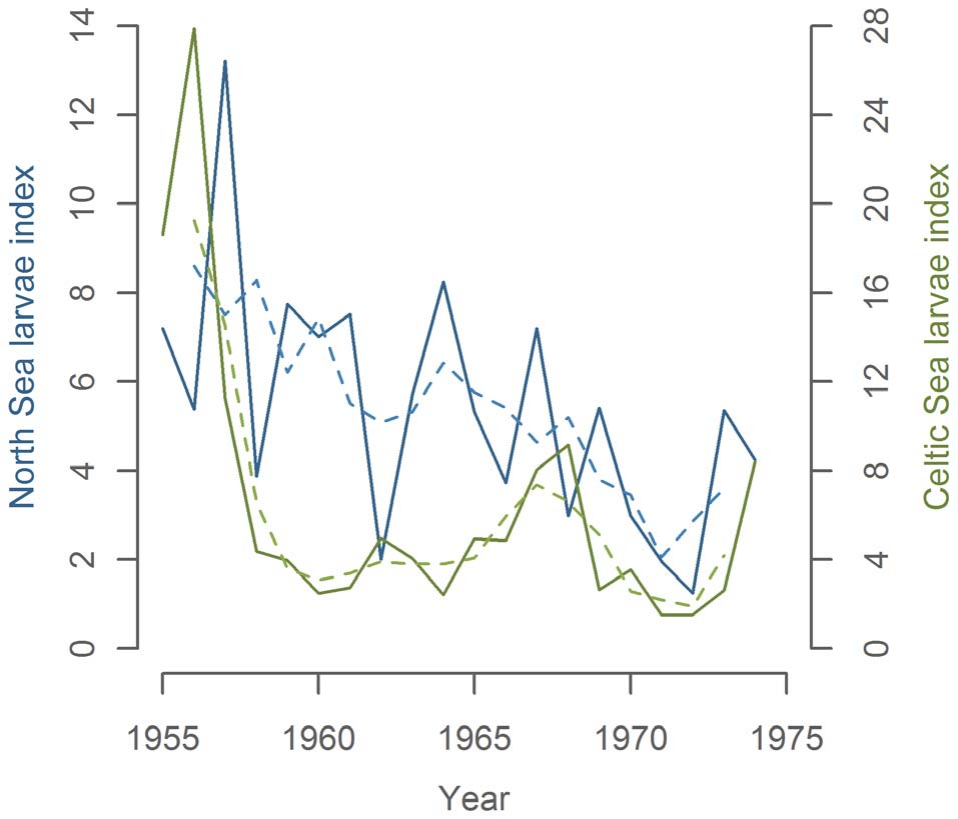
Mackerel larval indices in the North and Celtic Seas (full lines) with 3 year running mean trend lines (dashed lines).

The running means (rm) of larval indices in the North and Celtic seas were significantly positively correlated (3 year rm: R^2^ = 0.38, p = 0.007, Figure 3; 5 year rm: R^2^ = 0.33, p = 0.019), showing that the historical developments of the two stocks did not differ.

**Figure 4:**
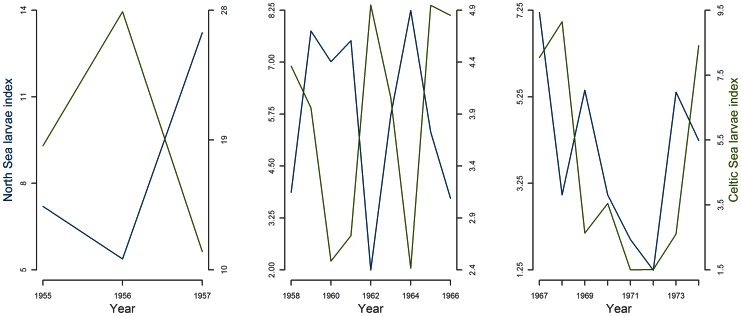
Mackerel larval indices in the North and Celtic Seas broken into three periods.

The detrended larval index in the North Sea in 1955–74 were negatively correlated with the larval index in the Celtic Sea (R^2^ = 0.23, p = 0.046) and so were the indices for the period 1958–1966 (R^2^ = 0.78, p = 0.004, Figure 4 middle).

The substantial interannual variability in this period 1958–1966 was higher in the North Sea (CV = 56%) than in the Celtic Sea (CV = 27%).

**Figure 5:**
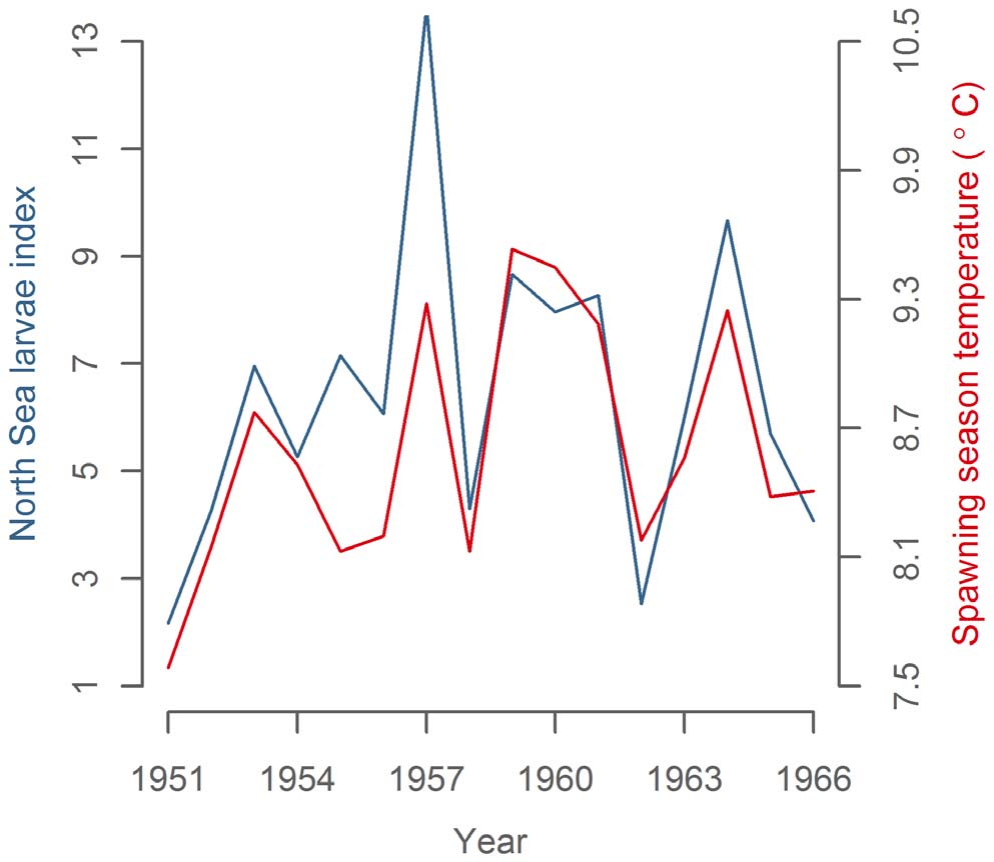
Mackerel larvae index from CPR surveys in the North Sea and sea surface temperature in the early spawning season (April-June) in the north-western North Sea (56–62°N, 0–4°W).

**Figure 6:**
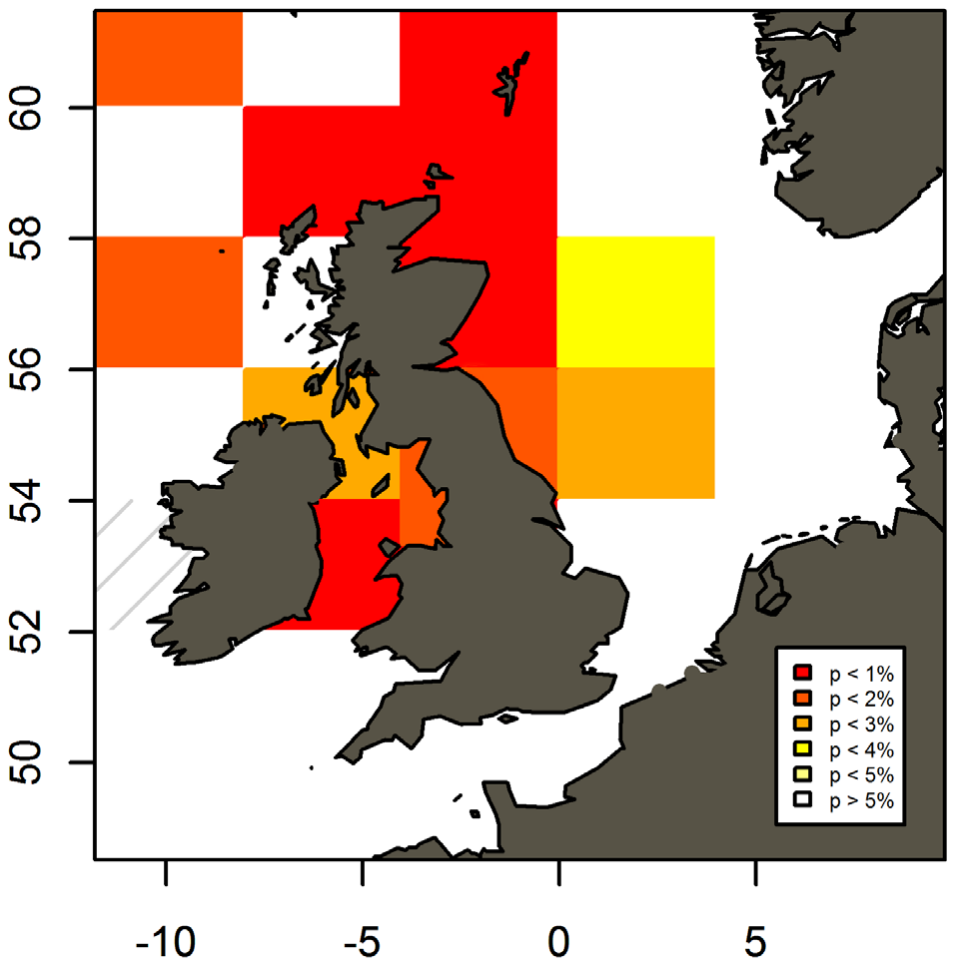
Spatial correlation patterns between the mackerel larvae index from CPR surveys in the North Sea and sea surface temperature in April–June. Stripes indicate areas with insufficient temperature observations (<6 years).

The only significant term in the final model of mackerel larvae was Spring_SST (R^2^ = 0.65, p<0.001, 56–62°N, 0–4°E, Figure 5). Maps of spatial correlation patterns between the index and Spring_SST (Figure 6) showed strong correlations in the current that is known to flow NE along the shelf edge from West to North of Scotland where it enters the North Sea through the Fair Isle channel and East of the Shetland Islands [64]. Comparable strong correlations were also found in this current as it continues SE along the Scottish East coast inside the North Sea. Weaker, but still significant, correlations were found in the central North Sea and Dogger area. SST in the Eastern North Sea, South of Dogger, the English Channel and the Celtic Sea were not significantly correlated to the North Sea larvae index.

### Age Distributions

**Figure 7:**
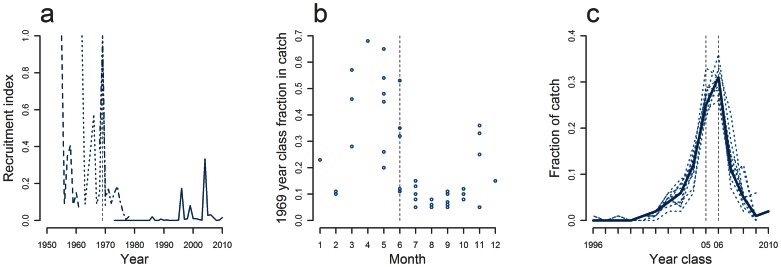
Age distributions and recruitment (a) Time series of recruitment in the North Sea indicating strong year classes. Please note that time series are not in scale. (b) Fraction of 1969 year class by month in 1974–1979 from commercial fisheries in the area between the Outer Hebrides and Cape Butt. (c) Age distributions in commercial catches in Jan-Mar 2010 by ICES division (stippled lines) and for all areas combined in Jul-Sep (bold line).

During bottom trawl surveys in the winter 1969–1970, unusual high numbers of juveniles were caught in the central North Sea, indicating massive recruitment inside the North Sea in 1969 [65]. The 1969 year class was the last of the large year classes in the 1960s (Figure 7a). Mackerel from this year class appeared in relatively large numbers in commercial catches all through the 1970s [63], [66]. To the north-west of Scotland, this strong year class was significant or dominant in the catches from March to May (Figure 7b). Later in summer the fraction of the catch belonging to the 1969 year class was much reduced, indicating that mackerel from the North Sea had either left the area and/or mackerel with another age distribution had entered. This has previously been interpreted as evidence for multiple stocks with different age compositions [63]. However, the relative decline of the dominance of the 1969 year class could also be explained by immigration of recruit spawners that spawn later than repeat spawners [67]–[69] and juveniles. This explanation cannot be ruled out because the complete age distributions were not published. However, it is more insightful to consider the fraction of the catch consisting of the 1969 year class in June, where it dominated in 3 out of 5 years. In these years mackerel from 1969 were relatively old and large repeat spawners of 5–10 years of age. Large repeat spawners are known to spawn early in the spawning season [67]–[69]. Spawning in the North Sea begins in May, peaks in late June or early July and ceases during July [51], [70]. Catch data from June 1974, 1976 and 1979 therefore indicate that mackerel originating from the North Sea was spawning outside the North Sea.

**Figure 8:**
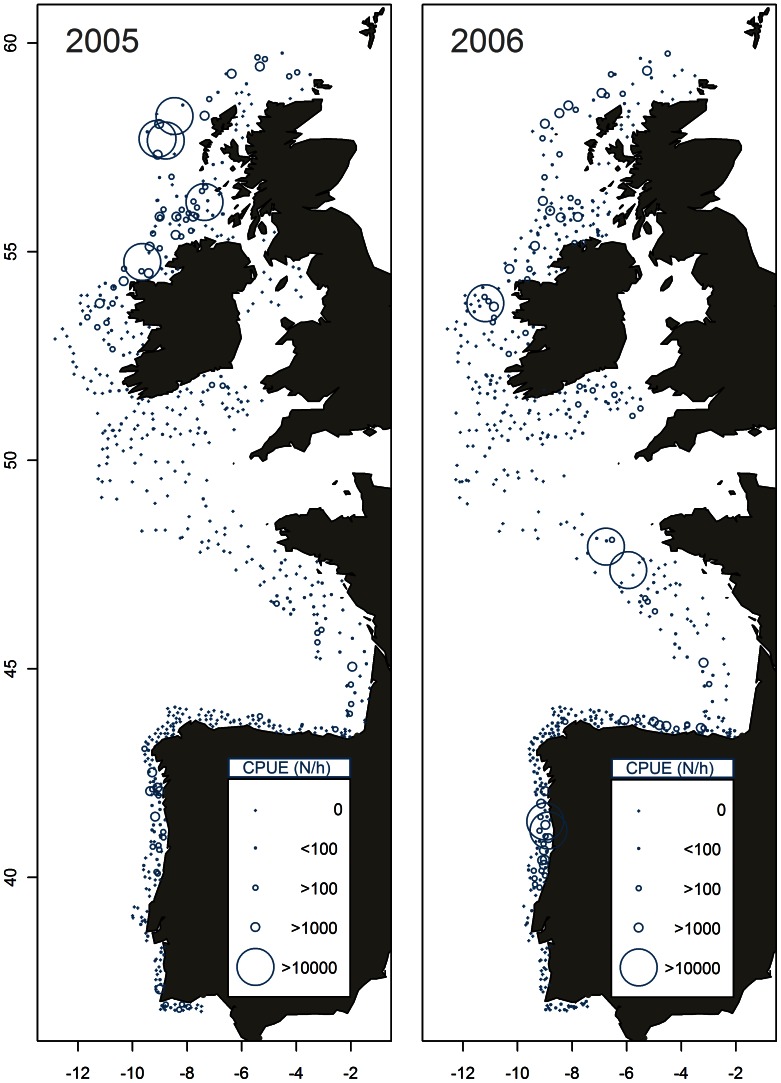
Spatial origin of recent strong year classes in the North East Atlantic. Catch Per Unit Effort (CPUE, numbers hour^−1^) of juvenile mackerel at the age of zero in October-December 2005 and 2006.

The exceptionally strong 2005 year class was, unlike other year classes such as the equally strong 2006 year class, primarily observed in the areas between Ireland and the Outer Hebrides (Figure 8). This indicates that the strong recruitment in 2005 was a spatially restricted event. As with the 1969 and 2006 year class, the strong 2005 year class was represented in the commercial catches in exceptionally high numbers in the subsequent years. This is apparent when inspecting catches from the main feeding season, when mixing between different components is assumed to be at its height (bold line on Figure 7c and table 2.4.1.1 in [1]). Since there are no substantial fisheries that target mackerel during spawning (table 2.4.1.1 [1]), it is only possible to compare data from different areas just prior to spawning. Age distributions of the catch in January - March from the Bay of Biscay in the south to the North Sea and waters around Scotland in the north, all have similar age compositions as the summer fisheries (Figure 7c). These age composition data thus indicate a substantial degree of straying between spawning components in recent years.

## Discussion

The analyses of mackerel spawning have demonstrated a significant negative relationship between larval densities in the North Sea and in the western spawning area. The similar stock trends with negative correlated interannual variability show that mackerel either switched spawning area preference from year to year or reacted oppositely to a common factor.

We found temperature to be highly correlated with the index, so this would be the prime candidate for the second explanation. However, since the larvae indices in both the North Sea and in the Celtic Sea were unrelated to the temperature in the Celtic Sea (Figure 6 and S1), we do not consider temperature related processes to be responsible for the observed patterns. Furthermore, if the pattern was caused by CPR catchability changes due to some large scale physical feature, we would expect to find the same pattern in the CPR time series for other species. No significant correlations were found between abundance time series of mackerel larvae and larvae of horse mackerel, clupeids, gobies, sandeels or dragonets (Unpublished data). Finally, we found that thermocline depth, which is likely to affect larval catchability, only affected the spatial dimension of the index *not* the interannual variation. It is also worth noting, that the water immediately behind a large, fast-moving vessel is likely to be mixed and homogenized well below the CPR towing depth [47]. The second explanation is therefore not the most likely explanation.

Therefore, we suggest that the positively correlated long term trends and especially the negatively correlated interannual variation indicate that the two spawning populations are connected by straying mackerel.

The analyses of age distributions supported the straying hypothesis by showing that strong year classes in some cases spread to other areas for spawning as adults. This phenomenon, known as “twinning”, is well-known for other species such as herring [14].

The analysis of the 2005 year class was based on commercial catch data without information on maturity stage, so we cannot be certain that the mackerel was spawning. However, the conclusions are robust to this uncertainty, because the spatial origin of the mackerel from the 2005 year class was very close to the main wintering areas [52]. If these mackerel were returning (homing) from the feeding areas, at similar or more northern latitudes, to spawn in the area west of Scotland, they would not be expected to pass through the Bay of Biscay. Because the 2005 year class dominated the catches throughout the Bay of Biscay at the peak spawning time in January to March 2010, we conclude that this demonstrate “twinning” i.e. substantial straying of this strong year class.

Similar support of straying was provided by the strong 1969 year class from the North Sea that seemed to spawn outside the North Sea. We consequently reject the null-hypothesis of reproductively isolated natal homing stocks in the North East Atlantic.

Atlantic mackerel clearly displays isolated and different life-cycle patterns across the Atlantic Ocean. On each side, there seems to be a complex of spatiotemporal diversity, but it is not evident that this diversity gives rise to isolated closed life cycle patterns i.e. contingents. The tendency for spatial segregation within the spawning migration in one of the traditional components shows that mackerel exhibits the necessary behavioral elements to generate closed spatiotemporal life cycle patterns on a more localized scale. However, no method has successfully been able to unequivocally identify the origin of spawning mackerel from any of the traditional spawning components. While most studies were found inconclusive, a weak phenotypic difference in the NWA indicated some structuring. On the other hand, the North Sea component, previously thought to be the most distinct component in the NEA, was found to mix into other spawning areas. Furthermore, a recent strong year class from West of Scotland now appears to have spread to other spawning areas.

The weak support for consistent structures and the indications of substantial mixing are mirrored by the genetics. On this basis, we suggest that the mackerel population in the NEA is best described as a dynamic cline, rather than as connected contingents. Temporal changes in hydrography and mackerel behavior may affect the steepness of the cline at various locations. A model that is able to simulate dynamic changes in return migrations and straying across the entire spawning area and in different seasons is needed in order to describe mackerel life-cycle pattern diversity in the NEA. However, such a model would need to be parameterized with data that are currently unavailable. Future effort should therefore be directed at monitoring techniques that can provide the needed rates of mixing and migration. Genetics, tagging and natural tracers (e.g. chemical, demographic, growth or parasites) have the potential to provide such data for mackerel as they have done for other species. These monitoring techniques should therefore be developed, standardized and implemented on a scale large enough to cover the mackerel life cycle.

It may be argued that Atlantic mackerel would meet the criteria for the strict definition of the metapopulation concept, sensu [10]. The criterion of more than one life-cycle pattern is clearly met by the isolation and differentiation across the Atlantic. Extinction is, like we argued for herring and cod, theoretically possibility e.g. on one side of the Atlantic Ocean.

However, under the assumption that mackerel in the North Western Atlantic are structured similarly to the mackerel in the North East, it is questionable how sensible it is to use the term “metapopulation” for Atlantic mackerel. It is not a “*population of local populations*” as Atlantic herring. We therefore recommend not to use the metapopulation concept to characterize the stock structure of Atlantic mackerel.

The hypothesis of North Sea mackerel as an isolated natal homing stock has been prevailing in mackerel science for half a century. A rejection of this hypothesis has implications for research, assessment and management of mackerel in the North East Atlantic. One consequence is that the history of the mackerel in the North Sea needs to be reviewed by expanding the single stock assessment techniques to account for migration dynamics and exchange with other spawning areas. This may lead to an improved understanding of the collapse as well as the lack of rebuilding.

The management of the mackerel fisheries in the northeast Atlantic has recently been severely challenged by changes in mackerel migration and distribution. In 2008 mackerel started to migrate into the economic zone of Iceland where a new fishery developed. This eventually led to the adoption of unilateral Icelandic and Faroese quotas and to a dispute about quota allocation with these two countries on one side, and the EU and Norway on the other. No solution has so far been reached and at the moment the total landings exceed the biologically recommended TAC. It has been shown that the incentive to reach a cooperative solution to a large extent will depend on the nature of the migrations [71], i.e. whether they result from random events, are density-dependent, or represent a permanent change in the distribution of mackerel. Our results point to a much greater flexibility in the migratory behaviour of mackerel than hitherto assumed. We consider the population of Northeast Atlantic mackerel to exhibit a cline of different genetic and behavioural adaptations generated by spatial segregation within the spawning migration and by straying, making it difficult to specify the optimal spatiotemporal pattern of fishing mortality from a biological point of view. However, while conservation of genetic and behavioural diversity is fundamental for sustainable fishing, it is clearly inadequate to use fixed geographical boundaries and historical rights to manage a highly migratory, dynamic and straddling fish stock.

Optimal management yielding the maximum sustainable yield within an ecosystem management context is likely to be compromised if the portfolio effect of diversity is reduced by a generalized management approach [72]. This should be of concern when managing mackerel fisheries, as some parts of the population may be overexploited. However, due to the substantial mixing along the cline, mackerel may seem less prone to unbalanced exploitation than many other commercial species. Additional tagging and modeling studies are needed to estimate the steepness of the clines on both sides of the Atlantic in order to specify the optimal spatiotemporal pattern of fishing mortality that will produce a sustainable catch without jeopardizing the genetic and behavioral diversity of the populations.

## Supporting Information

Figure S1 **Spatial correlation patterns between the mackerel larvae index from CPR surveys in the Celtic Sea and sea surface temperature in April-June.** Stripes indicate areas with insufficient temperature observations (<6 years).
(TIFF)
