# Supplementary figures and images for: Population Structure of Atlantic Mackerel (Scomber scombrus)
Source: PLoS One. 2013 May 31;8(5):e64744. doi: 10.1371/journal.pone.0064744 (PMC3669354; doi:10.1371/journal.pone.0064744)

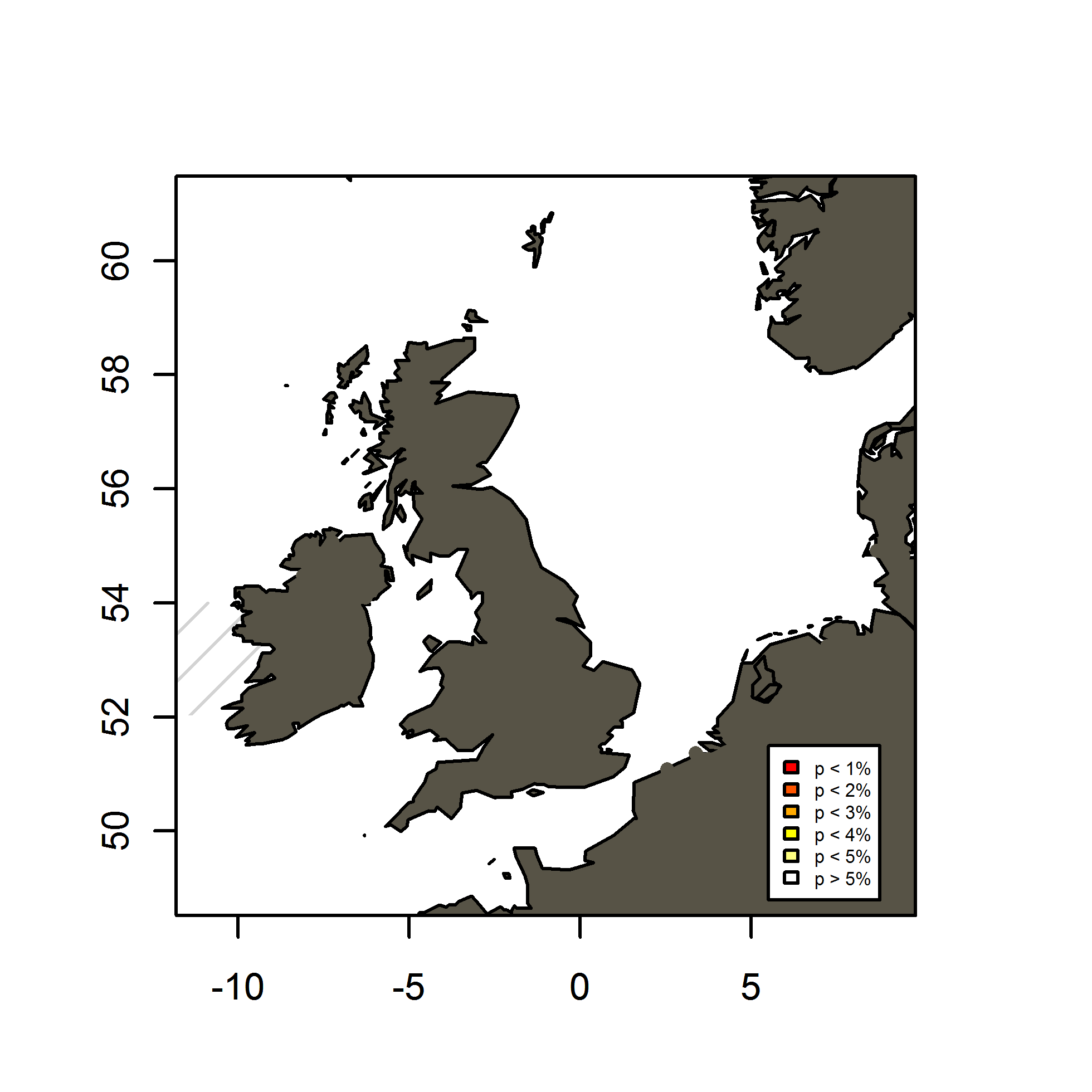

Supplement: Figure S1 — Spatial correlation patterns between the mackerel larvae index from CPR surveys in the Celtic Sea and sea surface temperature in April-June. Stripes indicate areas with insufficient temperature observations (<6 years). (TIFF) [file pone.0064744.s001.tiff]
